# The mechanism of bensulfuron-methyl complexation with β-cyclodextrin and 2-hydroxypropyl-β-cyclodextrin and effect on soil adsorption and bio-activity

**DOI:** 10.1038/s41598-018-38234-7

**Published:** 2019-02-13

**Authors:** Qianqian Geng, Tian Li, Xin Wang, Weijing Chu, Mengling Cai, Jingchan Xie, Hanwen Ni

**Affiliations:** College of Plant Protection, China Agricultural University, Beijing, P. R. China

## Abstract

In this work, the inclusion complexes of hydrophobic herbicide bensulfuron-methyl (BSM) with β-cyclodextrin (β-CD) and (2-hydroxypropyl)-β-CD (2-HP-β-CD) were prepared and characterized. Phase solubility study showed that both β-CD and 2-HP-β-CD increased the solubility of BSM. Three-dimensional structures of the inclusion complexes were simulated by the molecular docking method. The docking results indicated that guest BSM could enter into the cavities of host CDs, folded, and centrally aligned inside the inclusion complexes. The benzene ring of the guest molecule was close to the wide rim of the host molecules; the pyrimidine ring and side chains of the guest molecule were oriented toward the narrow rim of the host molecule. The inclusion complexes were successfully prepared by the coprecipitation method. The physiochemical characterization data of ^1^H NMR, FT-IR, XRD, and DSC showed that the guest and host molecules were well included. BSM had lower soil adsorption and higher herbicidal activity in the complexation form with β-CD or 2-HP-β-CD than in the pure form. The present study provides an approach to develop a novel CDs-based formulation for hydrophobic herbicides.

## Introduction

The use of herbicides has been an indispensable approach for controlling weeds and guaranteeing the yield of crops over the world^1,2^. Bensulfuron-methyl (methyl α-[(4, 6-dimethoxypyrimidin-2-ylcarbamoyl) sulfamoylmethyl]-o-toluat, BSM), one of sulfonylurea herbicides, is widely used as a pre-emergence herbicide to control broad-leaved weeds in rice paddy fields. It can be uptaken by weeds and translocated to target sites to inhibit the acetolactate synthase, block biosynthesis of branched-chain amino acids, and finally result in growth inhibition, chlorosis, and necrosis^3,4^. The solubility of BSM is very low (12–120 mg L^−1^ in water at pH 6.0–7.0 and 25 °C)^5,6^. It was reported that the herbicide was immobile to moderately mobile in soils and easy to be adsorbed by soil particles^6–8^. As BSM is a kind of soil-applied herbicide, the adsorption behavior has significant effect on its bioavailability, as well as on environmental fate^9,10^. There are many reports about its toxic effects to human^11^, phytoplankton^12^, aquatic animals^13^, and soil bacterial communities^14^. Reducing BSM application rate could decrease negative environmental effects and resistant weed risks. One of effective ways of reducing application rate and maintaining biological activity at the same time is increasing BSM solubility in water, which results in decreasing its adsorption by soil and increasing its bioavailability for weeds.

Cyclodextrins (CDs) are a class of water-soluble cyclic oligomers derived from starch, composed of six to eight D-glucose units linked by α-1,4-glucose bonds (often namely α-, β-, and γ-CD)^15,16^. The shape of CDs is just like a truncated cone with a hollow hydrophobic interior and a hydrophilic outer surface^17^. Due to this special structure, CDs can form inclusion complexes with a wide variety of organic molecules through the “host-guest interactions”: supramolecular self-assembly^18,19^. β-CD is most commonly employed and studied because it is cheap^17,20^. However, its application has some limitations due to its low solubility in water, so functional groups, such as hydroxyalkyl and methyl, are often introduced to parent CDs to overcome this shortcoming^21^. CDs and their derivatives are commonly used in food and pharmaceutical industries^22–25^. They are also used to remediate pesticide-contaminated environments and improve the formulation of pesticides^26,27^. Villaverde *et al*. found that β-CD could increase herbicide norflurazon desorption from soils^28^. However, some necessary data concerning the experimental and theoretical investigation of CDs complexation with hydrophobic herbicides, especially for the sulfonylurea herbicides, is still insufficient.

In the present study, the inclusion complexes of BSM with β-CD and 2-HP-β-CD were prepared. Molecular docking was conducted to get an insight into the inclusion behaviors between guest molecule BSM and host molecules β-CD and 2-HP-β-CD. The inclusion complexes were characterized by nuclear magnetic resonance (^1^H-NMR), Fourier transform infrared spectroscopy (FT-IR), powder X-ray diffraction (XRD), and differential scanning calorimetry (DSC) methods. What the authors most concerned was how BSM was included with β-CD and 2-HP-β-CD, weather its adsorption on paddy soil was decreased and its herbicidal activity was enhanced after complexation. This study would provide a potential approach for developing environment-friendly formulations with high water solubility, low soil adsorption, and high bio-activity for hydrophobic herbicides.

## Results and Discussion

### Phase solubility

**Figure 1:**
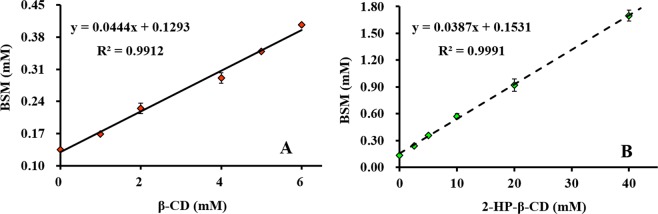
Phase solubility diagram of bensulfuron-methyl (BSM) with β-CD (**A**) and 2-HP-β-CD (**B**). (25 °C, pH = 6.5) Error bars are ± standard deviation, the error bars are shorter than the data labels occasionally. (n = 3).

The phase solubility diagrams of BSM with different concentrations of β-CD and 2-HP-β-CD were illustrated in Fig. 1. In the two phase solubility diagrams, BSM solubility increased linearly with the concentrations of the two CDs. Based on the theory of Higuchi and Connors^29^, these two diagrams could be classified as A_L_-type curves, which indicated that 1:1 stoichiometry of the inclusion complexes was formed between BSM and the two CDs. BSM solubility in water (25 °C, pH 6.5) increased from 55.2 mg L^−1^ up to 167.03 mg L^−1^ and 696.69 mg L^−1^, respectively, in β-CD and 2-HP-β-CD solutions. According to Loftsson *et al*.^25^, the apparent *Ks* of most inclusion complexes was ranged 50–2000 M^−1^. The calculated *Ks* values of the inclusion complexes of BSM/β-CD and BSM/2-HP-β-CD in this study were 316.6 and 277.6 M^−1^, respectively, which indicated that the inclusion tendency of guest BSM with host CDs was moderate. The *Ks* value of BSM/β-CD was bigger than that of BSM/2-HP-β-CD, which could be attributed to the steric hindrance caused by the substitution groups in 2-HP-β-CD. Similar results were reported about the inclusion complexes of luteolin and five CDs with different substitution groups^30^. The cavity of 2-HP-β-CD is bigger than that of β-CD so that the binding of BSM with 2-HP-β-CD is less stable than with β-CD. This is another reason why the apparent *Ks* of BSM/2-HP-β-CD was smaller than BSM/β-CD.

### Molecular docking

**Figure 2:**
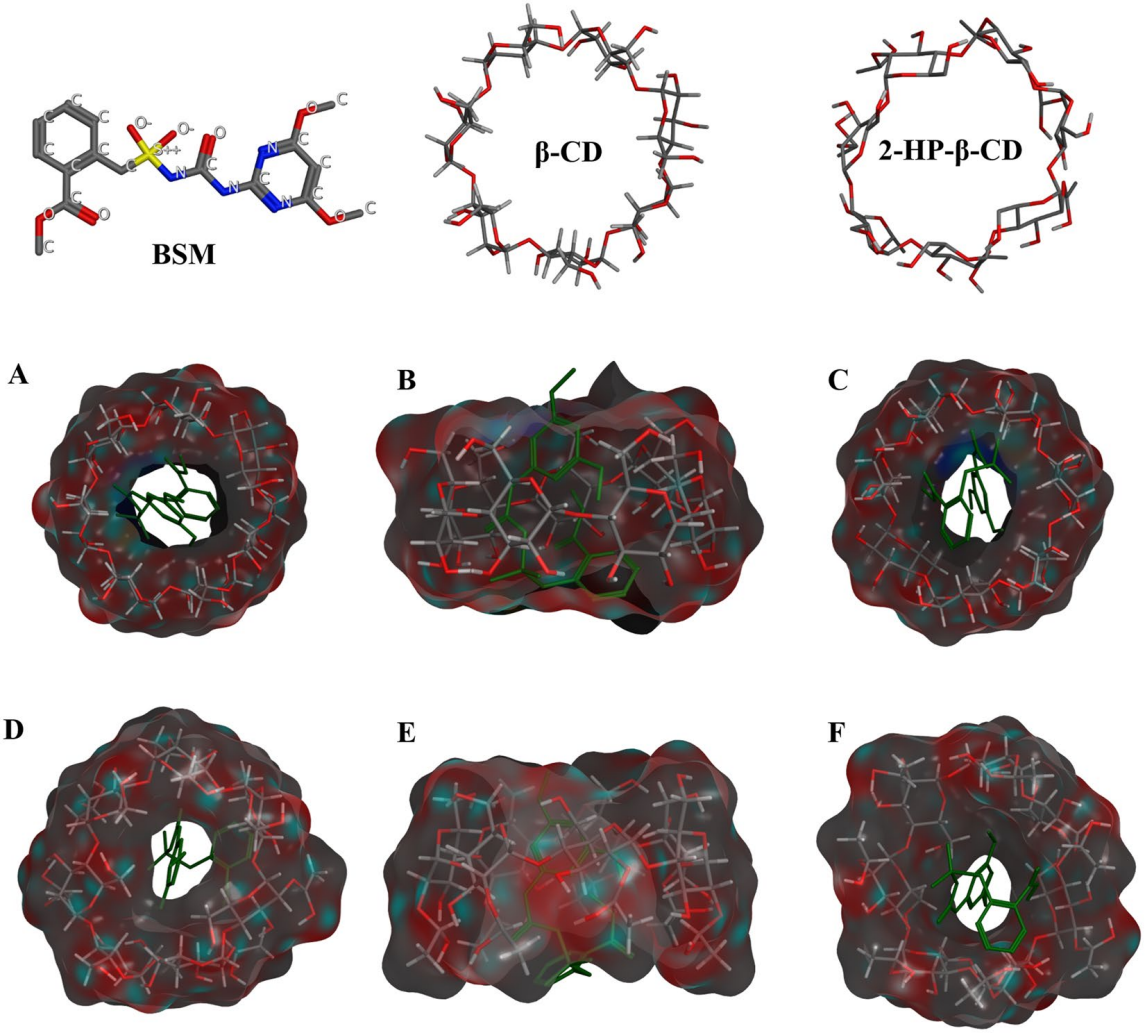
Optimized structures of BSM/β-CD (**A**) top view; (**B**) side view; (**C**) bottom view) and BSM/2-HP-β-CD (**D**) top view; (**E**) side view; (**F**) bottom view) inclusion complexes. BSM: bensulfuron-methyl, represented in green sticks, β-CD: β-cyclodextrin, 2-HP-β-CD: (2-hydroxypropyl)-β-cyclodextrin.

Molecular docking studies were adopted to elaborate the complexation mechanism of host and guest molecules in previous literatures^31–33^. In this work, the three-dimensional supermolecular structures of BSM/β-CD and BSM/2-HP-β-CD complexes were investigated using the docking method of Molecular Operating Environment (MOE). Based on the result of phase solubility, a stoichiometry of BSM and the CDs at the rate of 1:1 was chosen for molecular docking. Optimized structures of BSM/β-CD (A, top view; B, side view; C, bottom view) and BSM/2-HP-β-CD (D, top view; E, side view; F, bottom view) inclusion complexes were shown in Fig. 2. Molecular docking results indicated that guest BSM entered into the cavity of host CDs, folded, and centrally aligned inside them. The benzene ring of the guest molecule was close to the wide rim of host molecule. The pyrimidine ring and side chains of the guest molecule were oriented toward the narrow rim of the host molecule. The hollow inclusion space of β-CD was not enough to accommodate the entire molecule of BSM so that the alkyl chains of BSM were partly exposed (see Fig. 2). The binding energy of BSM/2-HP-β-CD (−15.399 kcal mol^−1^) was lower than that of BSM/β-CD (−12.966 kcal mol^−1^), which indicated that the inclusion capacity of β-CD was improved by the substitution of hydroxypropyl group. The reason for this could be that 2-HP-β-CD has larger cavity and hydrophobic pocket, which makes BSM enter easier into the cavity^29^, These molecular docking findings were confirmed by following NMR and FT-IR results.

### Physicochemical characterization of BSM/CDs complexes

#### NMR

**Figure 3:**
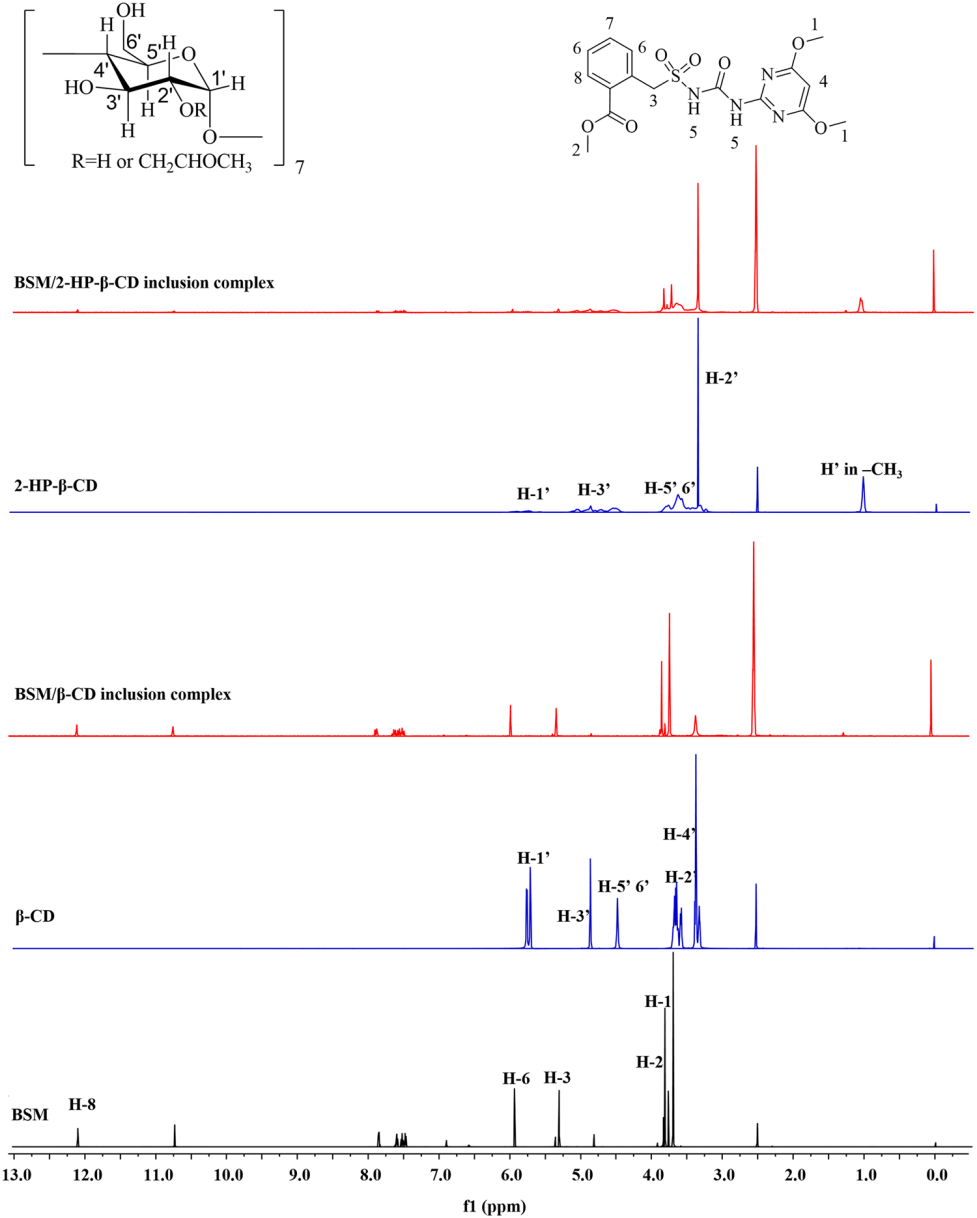
Proton nuclear magnetic resonance (^1^H NMR) spectra of bensulfuron-methyl (BSM), β-CD, 2-HP-β-CD, and corresponding inclusion complexes.

One of effective evidences for the formation of an inclusion complex is ^1^H NMR. Once a complex formed, the chemical shifts of both host and guest molecules would be influenced^33–35^. ^1^H NMR spectra of the host and guest molecules and corresponding complexes were compared (Fig. 3) and chemical shifts were listed in Table 1. The H protons of BSM showed distinct shifts in the presence of β-CD and 2-HP-β-CD, especially for H2, H3, and H5 protons, which indicated that BSM entered into the cavity of the CDs.

**Table 1:** 1H NMR chemical shift (Δδ) data of BSM/β-CD and BSM/2-HP-β-CD complexation.

H protons	ppm (DMSO-d6)				H protons	ppm (DMSO-d6)			
	BSM	β-CD	BSM/β-CD	Δδ^a^		BSM	HP- β-CD	BSM/ 2-HP-β-CD	Δδ^a^
H1	3.76	—	3.75	−0.01	H1	3.76	—	3.75	−0.01
H2	3.83	—	3.80	−0.03	H2	3.83	—	3.80	−0.03
H3	5.35	—	5.29	−0.06	H3	5.35	—	5.31	−0.04
H5	7.60	—	7.58	−0.02	H5	7.60	—	7.59	−0.01
H6	7.83	—	7.82	−0.01	H6	7.83	—	7.82	−0.01
H8	12.06	—	12.05	−0.01	H8	12.06	—	12.05	−0.01
H1′	—	5.72	ND		H1′	—	5.87	5.86	−0.01
H2′	—	3.62	ND		H2′	—	3.61	3.61	0.00
H3′	—	4.82	4.80	−0.02	H3′	—	4.83	4.80	−0.03
H4′	—	3.34	3.33	−0.01	H5′	—	3.74	3.75	0.01
H5′	—	4.45	ND		H6′	—	3.74	3.75	0.01
H6′	—	4.45	ND		CH3	—	1.02	1.02	0.00

^a^Δδ = δ (complex) - δ (free).

ND: not detected.

It was known that H-3′ was located inside the wide cavities of CDs and H-5′ and H-6′ located inside the narrow cavities, while H-2′ and H-4′ were located outside the cavities. The obtained data in Table 1 showed that H3′, H5′, and H6′ became shielded and had shifts in the presence of BSM, accompanied with the chemical shifts of the outer protons of H-2′ and H-4′. The chemical shift changes indicated the entrance of the guest molecule. In many previous reports, only the inside H protons of CDs, such as H3′ and H5′, were affected by the insert of guest molecules^36,37^. In this work, it was found that both the outer and inside H protons of the CDs got chemical shifts, including upfield, downfield shift, and shielded, which might be due to the joint effects of BSM inserting and the conformational rearrangement of the CDs after inserting. The results of ^1^H NMR spectrum studies and molecular docking were consistent.

#### FT-IR spectroscopy

**Figure 4:**
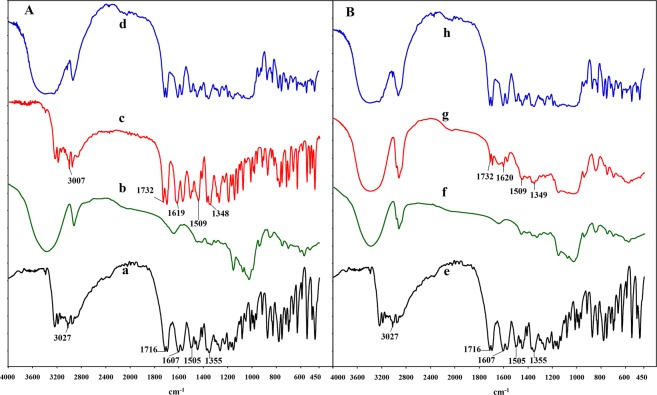
FT-IR spectra of (**A**) bensulfuron-methyl (BSM) (a), β-CD (b), BSM/β-CD inclusion complex (c), BSM + β-CD physical mixture (d); (**B**) bensulfuron-methyl (BSM) (e), 2-HP-β-CD (f), BSM/2-HP-β-CD inclusion complex (g), BSM + 2-HP-β-CD physical mixture (h).

FT-IR can provide enough information to confirm the formation of inclusion complexes based on the differences of the shape, shift, and intensity of absorption spectra^30,34^. FT-IR spectra of the individual host and guest molecules, their physical mixtures, and the inclusion complexes were presented in Fig. 4. As the existence of overlap between the host and guest molecules, not all the changes of FT-IR shifts could be observed in the complexes. As shown in Fig. 4A, BSM had five bands at 3027 cm^−1^, 1716 cm^−1^, 1607 cm^−1^, 1355 cm^−1^, and 1505 cm^−1^, which could be respectively assigned to the stretching frequencies of = C-H in phenyl, -C = O in phenyl substitution group, -C = N- bond in the pyrimidine nitrogen, and SO_2_ groups and bending vibration frequency of -NH (secondary amide group on the sulfonylurea bridge).

The spectra of physical mixtures of BSM with β-CD and 2-HP-β-CD were the superimposition of the individual absorption bands of BSM and the two CDs with no remarkable alterations. For the spectrum of BSM/β-CD complex (Fig. 4c), the above-mentioned five absorption bands of pure BSM were shifted to 3007 cm^−1^, 1732 cm^−1^, 1619 cm^−1^, 1348 cm^−1^, and 1509 cm^−1^, respectively, accompanied with intensity changes at different degrees. While in the spectrum of BSM/2-HP-β-CD complex (Fig. 4g), the stretching frequency of = C-H in phenyl disappeared, and the other four absorption bands held a similar shift change with those of BSM/β-CD (Fig. 4c). Based on these variations of the FT-IR absorption peaks, the benzene ring, pyrimidine ring, and sulfonylurea bridge of BSM were included into the cavity of the CDs and the inclusion complexes formed, which was in accordance with the results of molecular docking and NMR.

#### XRD

**Figure 5:**
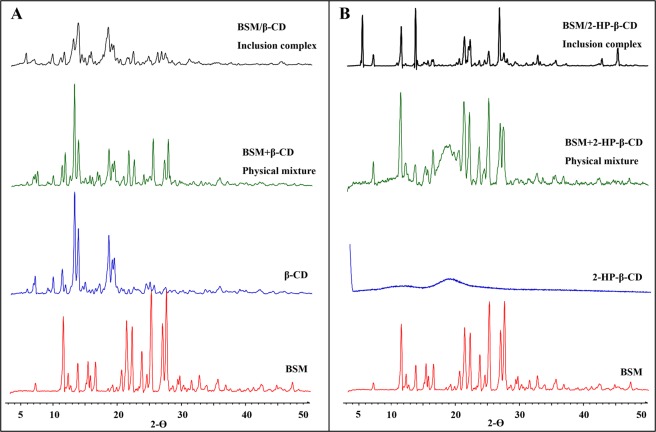
X-ray diffraction (XRD) patterns of bensulfuron-methyl (BSM) and their physical mixtures and inclusion complexes with (**A**) β-CD and (**B**) 2-HP-β-CD.

X-ray powder diffraction of pure BSM, β-CD, 2-HP-β-CD, the physical mixture of BSM with the CDs, and the inclusion complexes were given in Fig. 5. Sharp diffraction peaks indicated the high crystallinity of BSM and β-CD. The XRD pattern of BSM + β-CD physical mixture was similar with those of pure BSM and β-CD. The diffraction diagram obtained from the BSM/β-CD and BSM/2-HP-β-CD complexes showed a dramatic decrease in the number and intensity of diffraction peaks (e.g., compared with pure β-CD at 6.8°, 9.6°, 11.0°, 12.9°, 18.3°, 19.1° 2ϴ and pure BSM at 7.0°, 21.2°, 25.1°, 26.8°, 27.4° 2ϴ) and new peaks appeared at 26.5°, 27.1°, and 27.4° 2ϴ (Fig. 5A). The XRD diffraction spectrum of 2-HP-β-CD was smooth because it was in amorphous form. The XRD pattern of BSM + 2-HP-β-CD physical mixture was similar with that of BSM. There were similar changes of X-ray powder diffraction peaks in BSM/2-HP-β-CD complex (Fig. 5B). The diffraction diagram BSM/2-HP-β-CD complex also showed a dramatic decrease in the number and intensity of diffraction peaks at 15.2°, 16.4°, 21.2°, 22.1°, 23.6°, 25.1°, and 27.5° 2ϴ and new peaks appeared at 5.4° and 45.3° 2ϴ. These results showed that the crystalline structures of the inclusion complexes were different with that of pure BSM, which further confirmed the formation of the inclusion complex.

#### DSC measurement

**Figure 6:**
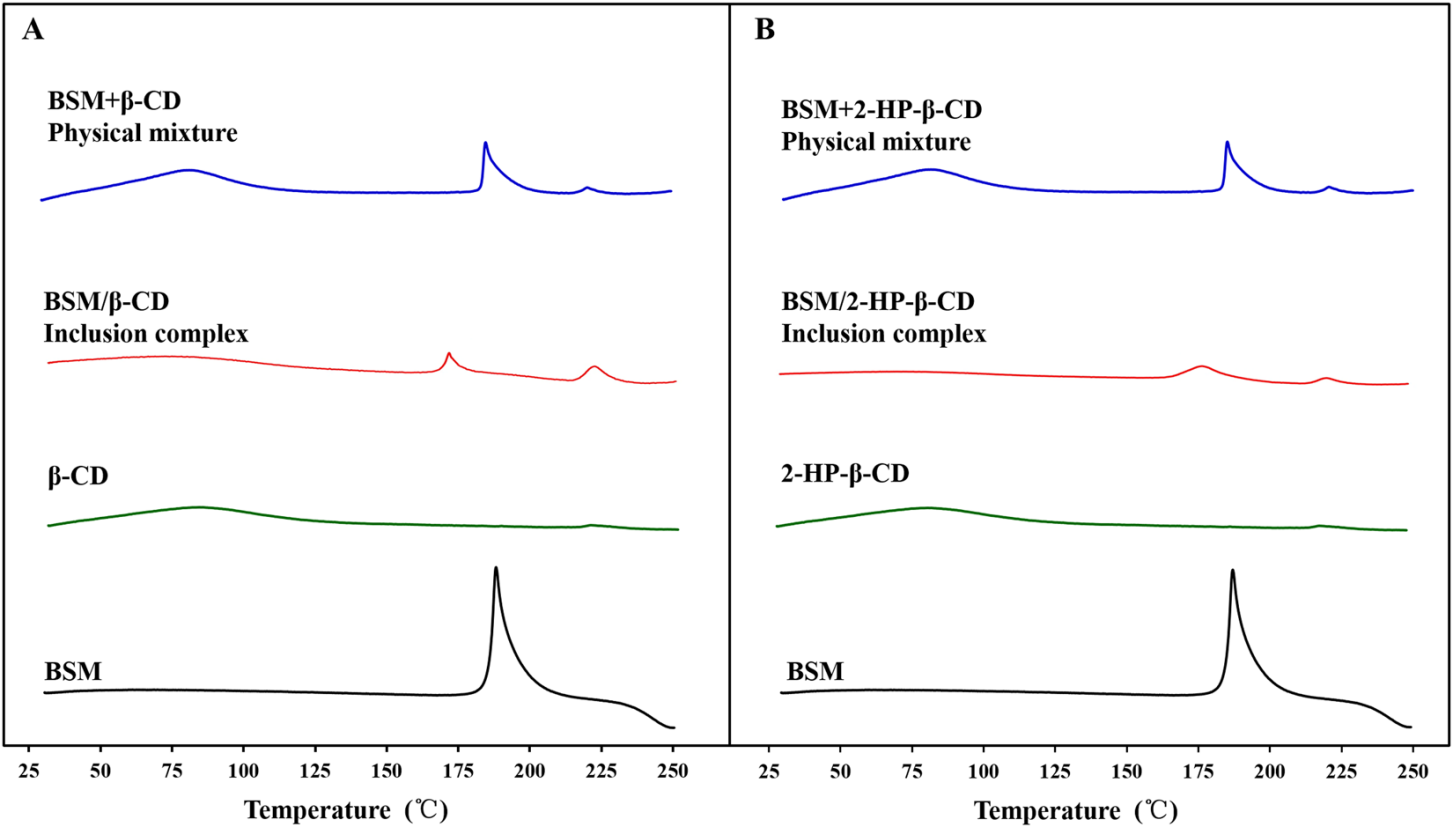
Differential scanning calorimetric (DSC) thermogram of bensulfuron-methyl (BSM) and their physical mixtures and inclusion complexes with β-CD (**A**) and 2-HP-β-CD (**B**).

DSC thermogram of BSM, the CDs, and their corresponding physical mixtures and inclusion complexes were illustrated in Fig. 6. DSC curve of BSM gave a characteristic endothermic fusion peak at about 190 °C. A broad and low endothermic peak was observed at 50–120 °C, which could be attributed to the liberation of crystal water from β-CD and 2-HP-β-CD. The DSC thermogram for the physical mixtures of BSM and the CDs had the same characteristic peaks of pure BSM and the CDs, which indicated that there was no interaction between BSM and the CDs in the physical mixtures. Compared with pure BSM, β-CD, 2-HP-β-CD and their physical mixtures, the DSC curves of BSM/β-CD and BSM/2-HP-β-CD complexes displayed completely different peak pattern: the endothermic fusion peak disappeared at 190 °C and new peaks appeared at 170 °C and 182 °C. The results indicated that strong interactions occurred between BSM and the CDs in their inclusion complexes. In addition, the dehydration curves in the two complexes decreased, which could be because of the substitution of water molecules by BSM in the CDs cavity and also indicated the formation of the inclusion complexes.

### Adsorption in paddy soil

**Figure 7:**
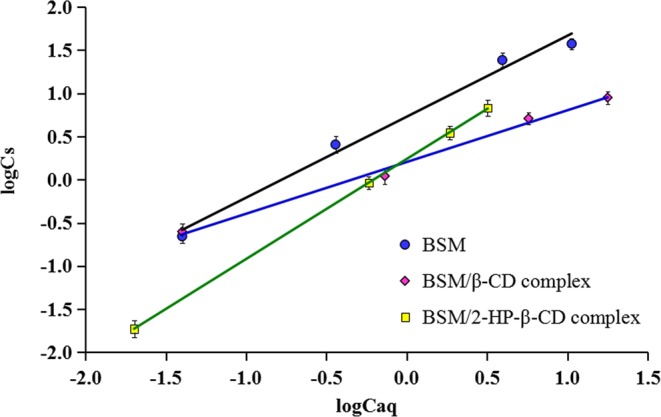
Freundlich adsorption isotherms of bensulfuron-methyl (BSM), BSM/β-CD complex BSM/2-HP-β-CD complex at different concentrations. *C*_*aq*_: concentration of bensulfuron-methyl in the aqueous phase (mg L^-1^); *C*_*s*_: concentration of bensulfuron-methyl in soil (mg kg^−1^).

The measured adsorption isotherms of pure BSM, BSM/β-CD complex, and BSM/2-HP-β-CD complex were found to be linear and could be well-fitted by Freundlich model (Fig. 7). The correlation coefficients of the three test materials were 0.9816, 0.9940, and 0.9999, respectively. The *N* values (the isotherm nonlinearity index) were all less than 1 (Table 2). According to the classification of Giles *et al*.^38^, all the adsorption isotherms were L-type, indicating that the BSM adsorption was concentration-dependent. This type adsorption of BSM and its inclusion complexes are due to the specific soil properties other than experiment artifacts^39^. The *N* value of BSM/2-HP-β-CD complex was bigger than those of pure BSM and BSM/β-CD complex, which might be because the substitution of hydroxypropyl changed the interaction between 2-HP-β-CD with the soil particles.

**Table 2:** Freundlich adsorption isotherm constants and characteristics derived from bensulfuron-methyl and its complexes with β-CD and 2-HP-β-CD.

Materials	K_f_^c^(mg^1−n^L^n^kg^−1^)^a^	N^c^	R^2^
BSM^b^	9.63 ± 2.12	0.59	0.9816
BSM/β-CD complex	1.77 ± 0.89	0.57	0.9940
BSM/2-HP-β-CD complex	1.69 ± 0.75	0.73	0.9999

^a^Mean values ± SD of three replicates.

^b^BSM: bensulfuron-methyl.

^c^*K*_*f*_ and *N* represent the binding constants and the isotherm nonlinearity index, respectively.

*K*_*f*_ value represents the adsorption capacity of a compound in soil. Based on the *K*_*f*_ values, the order of adsorption capacity was: BSM > BSM/β-CD > BSM/2-HP-β-CD, indicating the adsorption capacity of BSM was higher in the pure form than that in the inclusion complexes. The declined BSM adsorption might mainly contributed to water solubility enhancement by the CDs, which makes BSM be higher tendency to be retained in solution than to be adsorbed in the soil^26,28,40^. Bian *et al*.^40^ reported a similar result that the inclusion complex of herbicide butachlor with β-CD showed low adsorption potency in soil. BSM/2-HP-β-CD had lower adsorption than BSM/β-CD could be due to its much higher solubility and also might be because of their surface characteristics.

### Herbicidal activity

**Figure 8:**
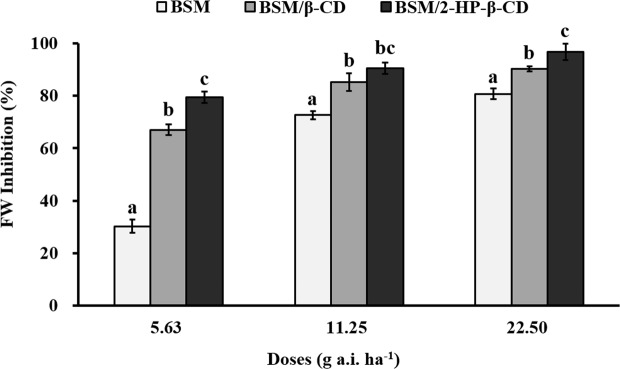
Herbicidal activities of bensulfuron-methyl (BSM) and its complexes with β-CD and 2-HP-β-CD on *Eclipta prostrata* in greenhouse. FW: fresh weight. Different letter denotes significant difference (p < 0.05) of the mean value among the treatments. Error bars are ±1 standard deviation (n = 5).

In the present experiment, effect of pure BSM, BSM/β-CD, and BSM/2-HP-β-CD on the growth of *Eclipta prostrata* was evaluated at different treatment doses in greenhouse. Their inhibition percentages were shown in Fig. 8. There was significant (p < 0.05) difference in growth inhibition between the inclusion complexes and pure BSM treatments at all the treated doses after 28 days application, and the biggest difference were at the low dose (5.63 g a.i. ha^−1^). The inhibition percentage for BSM/β-CD and BSM/2-HP-β-CD was 42.3% and 50.4% higher than that of pure BSM, respectively, at the low dose. The inhibition of BSM/2-HP-β-CD was significantly higher (p < 0.05) than that of BSM/β-CD at the doses of 5.63 and 22.50 g a.i. ha^−1^. The obtained data indicated that the inclusion complexes enhanced the herbicidal activities. The reason for this could possibly be that the high solubility and low soil adsorption of the BSM inclusion complexes gave more bioavailability of the herbicide for weeds, especially at the low dose.

## Conclusions

The inclusion complexes of BSM with β-CD and 2-HP-β-CD were prepared and characterized firstly. Phase solubility, molecular docking mode, and soil adsorption behavior of the complexes were investigated. The phase solubility experiment indicated that the BSM/β-CD and BSM/2-HP-β-CD complexes were formed by a molar ratio of 1:1 and the inclusion tendency of the guest BSM with the host CDs was moderate. BSM solubility increased with the increase of CDs concentrations; stable three-dimensional structures of inclusion complexes were obtained with the molecular docking simulation method. After that, the inclusion complexes of BSM/β-CD and BSM/2-HP-β-CD were successfully prepared by the coprecipitation method and then confirmed by ^1^H NMR, FT-IR, XRD, and DSC. Adsorption experiment results showed that the adsorption of BSM in paddy soil was decreased after complexation with the CDs. The obtained inclusion complexes displayed better herbicidal activities than pure BSM. The preparation of inclusion complexes with CDs would be a promising strategy to enhance water solubility, reduce soil adsorption, and enhance bio-activities for hydrophobic herbicides.

## Experimental

### Chemicals, regents and materials

BSM (≥97%) was provided by Nutrichem (Beijing, China). Both β-CD (98%, average M_W_ = 1135) and 2-HP-β-CD (98%, average M_W_ = 1541, DS = 7) were obtained from Aladdin Biochemical Technology (Shanghai, China) and used without further purification (Fig. 3). Acetonitrile of high-performance liquid chromatography (HPLC) grade was obtained from J.T. Baker (Phillipsburg, NJ, USA). Ultrapure water was prepared by a Milli-Q water purification system (Millipore, Billerica, MA, USA). All the other chemicals and regents were of analytical grade and purchased from Sinopharm Chemical Reagent (Beijing, China).

Soil samples were collected in the surface of a rice paddy field (0–20 cm depth) located in Beijing (E: 116°19′, N: 40°14′), air-dried, ground into powder using mortar pestle lightly, and then passed through a 60-mesh sieve before use. The physicochemical properties of the soil samples were as follows: pH, 7.37; cationic exchange capacity, 25.61 cmol kg^−1^; organic carbon, 20.4%; sand, 47.95%; silt, 30.75%; and clay, 21.30%.

Seeds of weed *E*. *prostrata* were collected in Beijing (E: 116°19′, N: 40°14′) and the surface was sterilized by sequentially washing with 2% sodium hypochlorite and sterilized water before use.

### Phase solubility test

The phase solubility studies were carried out according to Higuchi and Connors^41^. Briefly, 100 mg BSM (excess amount) was added to 25 mL of aqueous solutions containing increasing concentrations of β-CD (0–6.0 mM) and 2-HP-β-CD (0–40.0 mM). The suspensions were vigorously shaken in a HZQ-X300C thermostat shaker (Shanghai, China) at 25 °C for 48 h until the equilibrium attained. The samples were filtered through a 0.22-μm Millipore membrane filter and diluted to appropriate concentrations for determination. Three replicates were set for each treatment.

The phase solubility diagrams were drawn by plotting the equilibrium concentrations of BSM with corresponding CDs. The stability constant *Ks* was calculated based on the phase solubility diagrams following the equation^41^:

1 $$Ks={\rm{slope}}/{S}_{0}(1-{\rm{slope}})$$

where slope represents the slope of the phase solubility diagram, and *S*_0_ is the BSM equilibrium concentration in pure water without CDs.

### Molecular docking

MOE 2014.09 (Chemical Computing Group, Montreal, QC, Canada) was employed to simulate the possible binding mode of BSM with β-CD and 2-HP-β-CD. The 3D structures of β-CD (PDB ID: 3CGT) and BSM (PDB ID: 5FEM) were extracted from the data base of RCSB Protein Data Bank according to Schmidt *et al*.^42^ and Lonhienne *et al*.^43^, respectively. 2-HP-β-CD structure was built by adding 7 hydroxypropyl substituents to β-CD in position 2 using ChemBioDraw Ultral 12.0 molecular builder. Chemical structures were shown in Fig. 2. Energy minimization was conducted using “QuickPrep” protocol for both host CDs and guest BSM molecules with the MMFF94x force field. Docking was operated according to previous literature^29,44^. In the molecular docking section, the conformation of the guest molecule was flexible with rotatable chemical bonds and the host molecule was rigid. BSM was treated as the ligand and CDs were as the receptor. The binding site was selected with Site Finder function. Then the following docking was done according to the default docking protocol. The conformations of ligand were fitted with Triangle Matcher method. Finally, the inclusion complex was selected according to the docking energy and optimum scoring pose.

### Preparation of inclusion complex

The preparation of BSM/β-CD, 2-HP-β-CD inclusion complexes was conducted using coprecipitation method described in previous literature^45–47^. Briefly, β-CD of 1.7025 g and 2-HP-β-CD of 2.3123 g were accurately weighted and dissolved with 100 mL and 50 mL distilled water in a 250-mL flask, respectively. The concentration of both β-CD and 2-HP-β-CD solutions was 1.5 mmol. BSM (0.4104 g, 1.0 mmol) was added to the mixed solution of acetonitrile-acetone (2:1, v/v, 30 mL). After BSM was completely dissolved, the solution was dropped to the β-CD and 2-HP-β-CD solutions, respectively. Herein, excessive CDs were used to improve the complex yield. The mixed solution was stirred at room temperature for 48 h and kept at 4 °C for 12 h. Then the generated white precipitate was filtered and washed with minimum amount ethanol and water to remove uncomplexed BSM and β-CD. The resulting precipitate was then dried in a vacuum oven at 50 °C for 12 h. The obtained inclusion complexes were stored in a desiccator before further analysis. The 1:1 molar ratio physical mixtures of BSM and the CDs were prepared by mixing them directly in a stirrer for 5 min.

### HPLC analysis

An HPLC system (Shimadzu, Japan), equipped with two LC-20ATvp pumps and an SPD-20Avp ultraviolet detector, was applied to the quantitation of BSM. A reversed-phase Kromasil ODS C_18_ column (250  mm × 4.6 mm, 5 μm) linked with a guard column (Kromasil ODS C18, 4 mm × 3 mm) and Chromatograph Solution Light Chemstation for LC system were employed to record and process chromatographic data. HPLC method was referred to Zhou *et al*.^48^ with modification. The detection wavelength was 238 nm, the mobile phase was composed of acetonitrile and water (added 0.1% phosphorous) by 75/25 (v/v) with a flow rate of 1.0 mL min^−1^. Column was kept at 25 °C and the injection volume was 20 μL.

### NMR spectra

^1^H NMR spectra were acquired on a Bruker AvanceIII HD 700 MHz spectrometer (Beijing, China). In all ^1^H NMR experiments, DMSO was used as the solvent.

### FT-IR spectroscopy

The FT-IR spectra of BSM, β-CD, 2-HP-β-CD, their physical mixtures, and the inclusion complexes of BSM/β-CD and BSM/2-HP-β-CD were obtained by a Jasco FT-IR 5300 spectrophotometer using KBr pellets, and the wavelength ranging from 4000 cm^−1^ to 450 cm^−1^ with a resolution of 0.5 cm^−1^.

### XRD spectrum

The powder XRD spectrum was studied by a Bruker D8 Advance diffractometer Cu-K radiation (40 kV, 100 mA) at the scanning rate of 4° min^−1^. Powder samples were mounted on a vitreous sample holder and analyzed in the 2ϴ angle range of 3–50°.

### DSC measurement

DSC measurements were carried out with a STA 449F3 + ASC instrument (NETZSCH, Germany). About 4 mg sample was analyzed from 30–250 °C at a heating rate of 4 min^−1^ and a 60 mL min^−1^ dynamic nitrogen atmosphere.

### Adsorption experiment

Adsorption experiment was performed using a batch equilibration method according to Test Guideline of OPPTS 835.1230 (US EPA, 2008)^49^. Briefly, a volume of 25 mL CaCl_2_ solution (0.01 M) containing various concentrations (0.1, 1.0, 10.0, and 20.0 mg L^−1^) of BSM (calculated by the active ingredients) was added into 100-mL Teflon centrifuge tubes wrapped with aluminum foil, then 4.0 g soil was added in the tubes. The solutions were shaken at room temperature (25 ± 2 °C) for 48 h and then centrifuged at 1632 g for 20 min. The supernatant of 5 mL was transferred to a new tube and filtered using a 0.22-μm Millipore membrane filter. The concentration of BSM in the aqueous phase (*C*_*aq*_, mg L^−1^) was determined by HPLC described above. The adsorption isotherms were modeled by the empirical adsorption model of Freundlich equation:

2 $${C}_{s}={K}_{f}{{C}_{aq}}^{{\rm{N}}}$$

3 $$\mathrm{log}\,{C}_{s}=\,\mathrm{log}\,{K}_{f}+N\,\mathrm{log}\,{C}_{aq}$$

where *K*_*f*_ and *N* represent the binding constants and the isotherm nonlinearity index, respectively.

Two blank controls were set. One contained only soil (test compounds free) and the other contained only test compounds (soil free). Each treatment was replicated three times.

### Herbicidal activity tests

The pre-emergence activities of pure BSM, BSM/β-CD and BSM/2-HP-β-CD inclusion complex were evaluated in a greenhouse using a previously reported procedure^50^. The emulsions of pure BSM, BSM/β-CD, BSM/2-HP-β-CD inclusion complex were prepared according to Xu *et al*.^51^ and sprayed using a laboratory sprayer at 675 L ha^−1^. The paddy soil (about 200 g) in a plastic box (8.0 cm × 8.0 cm × 6 cm) was wetted with water. Sprouting seeds (10) of *E*. *prostrata* were sown and covered with the soil (0.5 cm depth) and sprayed with the test materials at 5.63, 11.25, and 22.50 g a.i. ha^−1^. Fresh weight was determined 28 days later and the percentage inhibition relative to the untreated control was calculated. There were five replicates for each treatment.

### Statistical analysis

Data were analyzed using SPSS software (version 23.0) as variance (one-way ANOVA) with Duncan test for multiple comparisons (p = 0.05).
